# A bibliometric insight into neoadjuvant chemotherapy in bladder cancer: trends, collaborations, and future avenues

**DOI:** 10.3389/fimmu.2024.1297542

**Published:** 2024-02-19

**Authors:** Yi Huang, Chengxiao Liao, Zefeng Shen, Yitong Zou, Weibin Xie, Qinghua Gan, Yuhui Yao, JunJiong Zheng, Jianqiu Kong

**Affiliations:** ^1^ Department of Urology, Sun Yat-Sen Memorial Hospital, Sun Yat-Sen University, Guangzhou, Guangdong, China; ^2^ Guangdong Provincial Key Laboratory of Malignant Tumor Epigenetics and Gene Regulation, Sun Yat-Sen Memorial Hospital, Sun Yat-Sen University, Guangzhou, Guangdong, China; ^3^ Department of Urology, Peking University Shenzhen Hospital, Shenzhen, Guangdong, China

**Keywords:** bladder cancer, neoadjuvant chemotherapy, immune checkpoint inhibitors, platin-based, bibliometrics

## Abstract

**Background:**

Neoadjuvant chemotherapy (NAC) followed by radical cystectomy (RC) remains the cornerstone of treatment for muscle-invasive bladder cancer (MIBC). While platinum-based regimens have demonstrated benefits in tumor downstaging and improved long-term survival for selected patients, they may pose risks for those who are ineligible or unresponsive to chemotherapy.

**Objective:**

We undertook a bibliometric analysis to elucidate the breadth of literature on NAC in bladder cancer, discern research trajectories, and underscore emerging avenues of investigation.

**Methods:**

A systematic search of the Web of Science Core Collection (WoSCC) was conducted to identify articles pertaining to NAC in bladder cancer from 1999 to 2022. Advanced bibliometric tools, such as VOSviewer, CiteSpace, and SCImago Graphica, facilitated the examination and depicted the publication trends, geographic contributions, institutional affiliations, journal prominence, author collaborations, and salient keywords, emphasizing the top 25 citation bursts.

**Results:**

Our analysis included 1836 publications spanning 1999 to 2022, indicating a growing trend in both annual publications and citations related to NAC in bladder cancer. The United States emerged as the predominant contributor in terms of publications, citations, and international collaborations. The University of Texas was the leading institution in publication output. “Urologic Oncology Seminars and Original Investigations” was the primary publishing journal, while “European Urology” boasted the highest impact factor. Shariat, Shahrokh F., and Grossman, H.B., were identified as the most prolific and co-cited authors, respectively. Keyword analysis revealed both frequency of occurrence and citation bursts, highlighting areas of concentrated study. Notably, the integration of immunochemotherapy is projected to experience substantial growth in forthcoming research.

**Conclusions:**

Our bibliometric assessment provides a panoramic view of the research milieu surrounding neoadjuvant chemotherapy for bladder cancer, encapsulating the present state, evolving trends, and potential future directions, with a particular emphasis on the promise of immunochemotherapy.

## Introduction

Bladder cancer ranks as the 10th most prevalent cancer globally and represents the most frequently diagnosed malignancy within the urinary system (1). According to the latest global cancer statistics, bladder cancer accounts for approximately 3% of all cancer cases worldwide, emphasizing its significant impact on global health (2). It is categorized, based on pathological or radiological evaluations, into Non-Muscle Invasive Bladder Cancer (NMIBC) and Muscle Invasive Bladder Cancer (MIBC). MIBC patients exhibit a markedly heightened risk of recurrence and lymphatic metastasis relative to those with NMIBC. Sole reliance on surgical resection for treatment is associated with suboptimal therapeutic outcomes, with recurrence rates in lymph node-positive patients reaching up to 76% (3, 4).

To address this clinical challenge, perioperative treatments, including neoadjuvant and adjuvant therapies, have been rigorously explored to enhance patient outcomes. Introduced in the 1980s, neoadjuvant chemotherapy has been integrated into modern clinical paradigms (5, 6). This regimen seeks to reduce primary tumor size, mitigate remote micro-metastasis, and bolster curative potential preceding surgery. Contemporary recommendations endorse cisplatin-based combination regimens for eligible patients, delivering a modest but notable 5-8% uptick in overall survival (OS) (7, 8). Nevertheless, the broader application of chemotherapy is hindered by its toxicity profile and the diverse nature of bladder cancer.

The advent of high-throughput genomic sequencing technologies, such as next-generation sequencing (NGS), has revolutionized bladder cancer research (9). These advancements have enabled a detailed understanding of the genetic landscape of bladder cancer, identifying critical mutations in genes like FGFR3, TP53, and RB1. Such discoveries are instrumental in developing targeted therapies and predicting patient responses to treatments like neoadjuvant chemotherapy (10). Furthermore, genomic sequencing has facilitated the identification of molecular subtypes of bladder cancer, providing insights into their distinct prognostic and therapeutic implications. For instance, recent studies have shown that certain genomic alterations are associated with a better response to cisplatin-based chemotherapy, guiding treatment decisions and improving patient outcomes (11). Recent advances in immunotherapeutic interventions have been remarkable, with several agents undergoing clinical evaluation and manifesting encouraging therapeutic profiles. Immune checkpoint inhibitors (ICIs), particularly those targeting PD-1 or PD-L1 pathways, such as pembrolizumab, have demonstrated significant efficacy in advanced bladder cancer, especially when combined with chemotherapy (12, 13). The integration of genomic data with immunotherapy research is poised to further revolutionize the therapeutic landscape of bladder cancer, offering more personalized and effective treatment options (10). In the contemporary landscape of bladder cancer research, characterized by an ever-expanding corpus of scholarly articles. Conventional review methodologies, while undeniably valuable, often grapple with the formidable challenge of comprehensively elucidating the swiftly evolving sphere of neoadjuvant chemotherapy for bladder cancer.

Bibliometrics, a statistical approach, furnishes an analytical perspective on publications, presenting a methodical depiction of research trajectories and emphases. Through mathematical techniques, it illuminates various facets of scholarly contributions, including affiliations, journals, authors, citations, and keywords (14, 15). The advent of visualization platforms, like CiteSpace and VOSviewer, has facilitated the intuitive exploration and visualization of interrelationships based on publication, citation, and collaboration metrics sourced from open databases (16, 17). In this study, we will utilize advanced bibliometric techniques to assess the current landscape of neoadjuvant chemotherapy research for bladder cancer. By doing so, we seek to illuminate the most influential works, prominent research clusters, and emerging trends in the field. Furthermore, our analysis aims to provide researchers and clinicians with a clear roadmap of the domain’s evolution and to pinpoint areas that warrant further exploration.

## Methods

### Data source and acquisition

We searched the Clarivate Analytics Web of Science Core Collection database (WoSCC), which is one of the most commonly used repositories for bibliometric analysis, to identify and collect related publications on neoadjuvant chemotherapy of bladder cancer. To refine our search, we included articles that specifically mentioned “neoadjuvant chemotherapy” in the title, abstract, or keywords, and were related to bladder cancer, ensuring focused relevance. We excluded conference abstracts, editorials, and letters, aiming for a comprehensive collection of original research articles and reviews. In an effort to mitigate potential biases associated with updates to articles, the search was jointly executed by two independent researchers and was finalized on June 16, 2023.

### Search strategy

The employed search algorithm was delineated as: topic = (neoadjuvant or NAC) NEAR/1 (therapy* OR treatment* OR chemotherapy*) AND topic = (bladder) NEAR/1 (cancer* OR tumor* OR tumour* OR oncology OR neoplasm* OR carcinoma*) with a publication year constraint of 1999-2022. This query yielded 2549 articles within the WoSCC database. To ensure relevance and quality, we manually reviewed the titles and abstracts of these articles, selecting those that directly addressed neoadjuvant chemotherapy in the context of bladder cancer. Articles that were not focused on this specific topic or did not provide significant insights into the field were excluded. We further refined our selection to English-language articles and reviews from the specified date range, culminating in 1836 publications for subsequent analysis. Ethical approval was deemed non-essential for this study.

### Bibliometric analysis tools

For our bibliometric analyses, we utilized SCImago Graphica (Beta 1.0.35), Citespace (version 5.8.R2), and VOSviewer (version 1.6.16).

### Visualization and data presentation

In our bibliometric analysis, we employed several mathematical techniques to extract meaningful patterns and trends from the data. One key technique was the use of frequency and co-occurrence analysis, which helped in identifying the most frequently addressed topics and the relationships between different concepts within the literature. We also applied cluster analysis to group related articles, authors, or keywords into distinct clusters, revealing thematic concentrations and patterns of research activity. Network analysis was another pivotal technique, where we constructed and analyzed networks of citations and co-authorships to uncover the most influential authors and publications, as well as to visualize the collaborative landscape. We incorporated temporal analysis to trace the evolution of research themes over time, thereby identifying both historical and emerging trends (18).

To assist readers in interpreting these visualizations, we have added a brief tutorial. For instance, in CiteSpace, each node represents an article, author, or keyword, with the size of the node corresponding to its citation count or occurrence frequency. The lines between nodes represent co-citations or co-occurrences, indicating thematic or collaborative connections (19). Similarly, in VOSviewer, network maps display clusters of related items, with different colors representing different clusters. The distance between nodes in these maps reflects the strength of the relationship, with closer nodes indicating stronger connections.

Furthermore, we present illustrative examples to demonstrate the practical application of our findings. For example, one of our network maps highlights a significant cluster of research focused on the role of PD-1/PD-L1 inhibitors in neoadjuvant chemotherapy for bladder cancer. This cluster includes key publications and influential authors, illustrating the growing interest and pivotal research in this area. Another example is our temporal analysis graph, which shows the evolution of research themes over time, highlighting the shift from traditional chemotherapy approaches to combined chemo-immunotherapy regimens in recent years.

Citespace facilitated the extraction of annual publication and citation graphs, country collaboration diagrams, dual-map journal overlays, and the 25 most salient keywords based on citation bursts. Both VOSviewer and SCImago Graphica were instrumental in generating graphs depicting collaborative ties between nations, institutions, and authors, in addition to keyword analyses. Microsoft Office Excel 2019 (Microsoft, Redmond, WA, USA) was employed to illustrate publication trends and project anticipated publication counts for the ensuing year. Within our bibliometric analysis, the H-index of journals was calculated following the method proposed by Hirsch JE (20). This index is determined by arranging the published articles of a journal in descending order of their citation counts, and identifying the number “H”, where at least “H” articles have received “H” or more citations each.

## Result

### Annual publications trend

**Figure 1:**
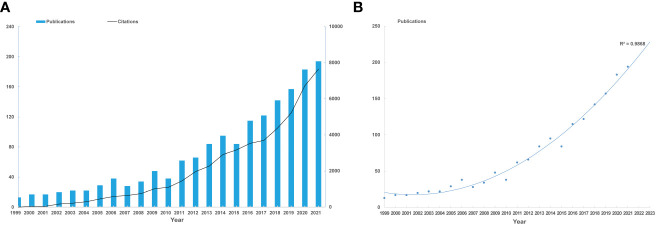
Annual trends of global publications and citations. **(A)** Numbers of publications and citations on NAC for bladder cancer from 1999 to 2021. **(B)** Polynomial fitting curve of annual publication in NAC for bladder cancer.

Our bibliometric analysis encompassed 1,836 publications from 1999 to 2022. Original research articles constituted 78% of these, and comprehensive reviews, 22%. Yearly publication output and citations showed a consistent increase, with a zenith in 2021 (**Figure 1A**). From 2012 to 2022, annual publication output doubled. A polynomial fitting curve (**Figure 1B**) was employed to elucidate this trend. The analysis rendered a coefficient of determination (R²=0.9868), emphasizing the trend’s statistical significance. Projections suggest approximately 230 publications in 2023. These data highlight the escalating interest in NAC for bladder cancer.

### Countries/regions

**Figure 2:**
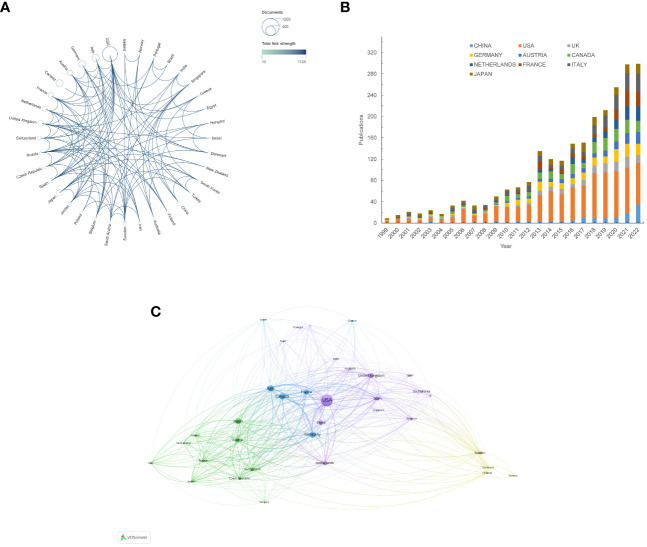
Analysis of global research trends and collaborations. **(A)** The total link strength of publications in different countries/regions. **(B)** The composition of annual publications in terms of the top 10 productive countries/regions. **(C)** Visualization graph of collaborations between countries/regions in NAC for bladder cancer. the sizes of the bubbles bearing the names of countries correspond to the respective quantities of publications, while the color of the bubbles represents the total link strength (TLS). Connection lines between countries denote cooperation relationships.

Researcher from 57 countries or regions participated in the study of NAC for bladder cancer in total. **Table 1** provides a comprehensive overview of the top 10 most productive nations in this research field. Notably, Europe emerges as a dominant contributor, with six countries, while North America and Asia each account for two. The United States stands out prominently, boasting the highest number of publications, total citations, and an impressive H-index of 98, which indicates academic output and quality. This conveys the United States’ indisputable leadership in the study of NAC for bladder cancer (**Figure 2A**). Furthermore, the United Kingdom garners distinction by achieving the highest average citation per paper, indicative of the nation’s production of high-quality studies with a notable influence on average. It is noteworthy that China, the sole developing country among the top 10, has demonstrated remarkable growth. Over the past five years, China has witnessed a two-fold increase in the quantity of publications (**Figure 2B**). A collaboration map (**Figure 2C**) depicts the US as the nexus of international collaborations, with North America and Europe as major participants.

**Table 1:** Top 10 productive countries/regions involved in neoadjuvant chemotherapy for bladder cancer.

Rank	Country	Counts	H-index	Total citations	Average citation per paper
1	United States	929	98	37865	40.76
2	Italy	230	45	8261	35.83
3	Canada	224	49	9311	41.57
4	Germany	209	46	11288	54.01
5	Japan	184	32	4378	23.79
6	United Kingdom	160	42	10238	61.69
7	France	150	32	6604	44.03
8	Netherlands	132	34	6695	50.72
9	Austria	125	33	6157	49.26
10	China	124	20	1219	10.98

### Institutes

As shown in **Table 2**, a detailed analysis of the top 10 prolific institutes in NAC research for bladder cancer highlights a significant presence of institutions from North America and Europe. Specifically, five of these top institutes are based in the United States, two in Canada, and the remaining three in Austria, Germany, and Italy. Within this competitive landscape, the University of Texas emerges as the most prolific institution, contributing a substantial number of papers to this field. Meanwhile, Memorial Sloan-Kettering Cancer Center stands out by accruing the highest number of total citations, indicative of its significant impact on the research domain.

**Table 2:** Top 10 institutes involved in neoadjuvant chemotherapy for bladder cancer.

Rank	Institutions	Countries/regions	Count	TLS	Total citations
1	University Texas	United States	104	239	6300
2	Memorial Sloan-Kettering cancer center	United States	91	182	6740
3	University of Vienna	Austria	85	371	3257
4	University of Montreal	Canada	58	259	3580
5	University of Hamburg	Germany	54	222	1423
6	Baylor college of Medicine	United States	53	171	4347
7	Cornell University	United States	52	260	1350
8	Mcgill University	Canada	51	179	2830
9	Mayo Clinic	United States	50	59	1508
10	Vita-salute San Raffaele University	Italy	47	171	1556

TLS, total link strength.

**Figure 3:**
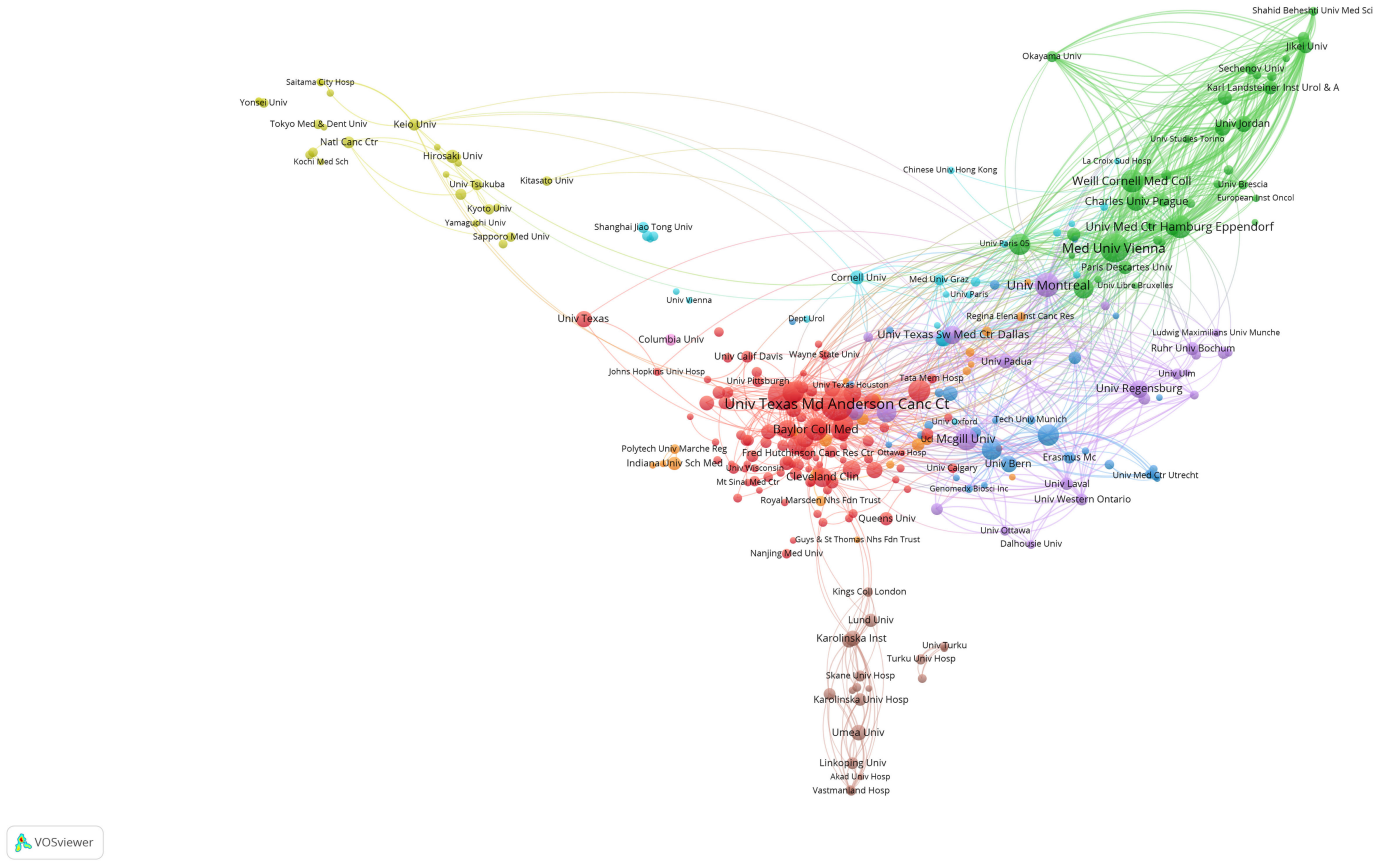
Visualization graph of collaborations between institutes in NAC for bladder cancer with a minimum of 5 documents. Each node represents an institute, with node size indicating their publication volume, and the lines between institutions representing their collaborations.

A visual representation of institutional collaborations, as depicted in **Figure 3**, gives the noteworthy observation that collaborations are predominantly fostered between American and European institutions. This intercontinental collaborative endeavor reflects the global nature of research in NAC for bladder cancer, with North American and European institutions playing pivotal roles in facilitating these cooperative relationships.

### Journals and co-cited journals

In **Table 3**, we present a comprehensive overview of the top 10 journals pertinent to NAC for bladder cancer. Strikingly, five of these journals originate from the United States, while two are affiliated with institutions in the Netherlands. The journals’ impact factors vary, ranging from a peak of 23.4 to a minimum of 1.1. European Urology claims the distinction of the highest impact factor, standing at 23.4, and additionally boasts the most total citations, tallying an impressive 12,137. This underscores the journal’s remarkable academic influence within the domain of NAC for bladder cancer. Intriguingly, despite Urologic Oncology-Seminars and Original Investigations publishing the highest volume of papers, it garners 2,231 total citations and possesses an influence factor of 2.7. This delineates a crucial point that in academic impact, both quantity and quality of publications wield significance. Also we calculated the H-indices of key journals in the field of urologic oncology. Notably, “The Journal of Urology” and “Cancer” emerged as leading publications with H-indices of 236 and 277, respectively. This contrasts with “Bladder Cancer”, a relatively newer journal, which currently has an H-index of 0. These indices reflect the varying levels of citation impact and scholarly influence among these publications in the field. Furthermore, eight out of the top 10 journals are specialized in urology, while the remaining two predominantly focus on oncology.

**Table 3:** Top 10 Journals related to the research of neoadjuvant chemotherapy for bladder cancer.

Rank	Journal title	Countries	Count	IF (2022)	JCR (2022)	TLS	Total citations	H-index
1	Urologic oncology-seminars and original investigations	United States	142	2.700	Q2	1323	2231	62
2	Journal of Urology	United States	111	6.600	Q1	1870	6718	236
3	BJU International	England	96	4.500	Q1	1054	3092	133
4	European Urology	Netherlands	87	23.400	Q1	2764	12137	187
5	World Journal of Urology	Germany	85	3.400	Q2	874	1095	75
6	Clinical Genitourinary Cancer	United States	78	3.200	Q2	844	919	40
7	Urology	United States	52	2.100	Q3	688	1645	165
8	Cancer	United States	36	6.200	Q1	912	2599	277
9	Bladder Cancer	Netherlands	31	1.100	Q4	385	237	0
10	Expert Review of Anticancer Therapy	England	30	3.300	Q3	315	239	59

TLS, total link strength.

**Figure 4:**
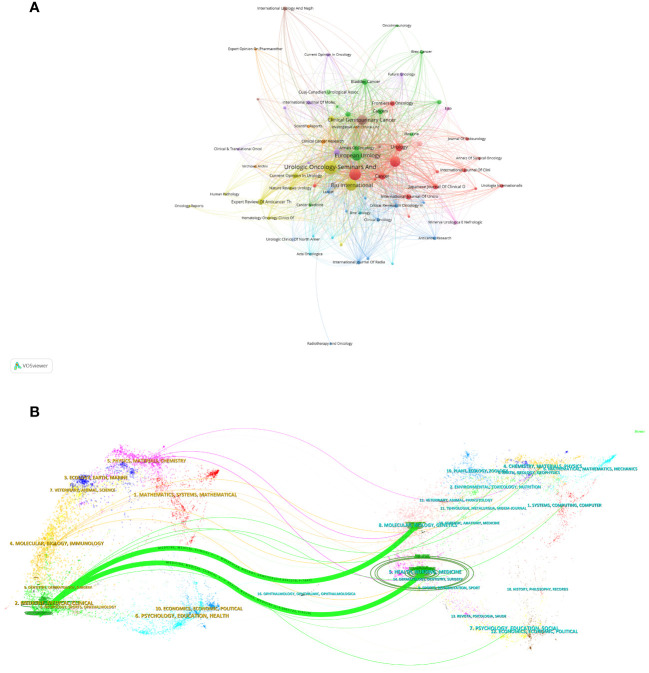
Visualization graph of collaborations between journals in NAC for bladder cancer with a minimum of 5 documents. Each node represents a journal, with node size indicating their publication volume, and the lines between journals representing their collaborations **(A)**. **(B)** The dual-map overlay of journals related to NAC for bladder cancer. The citing journals were on the left, the cited journals were on the right, and the colored path represents the citation relationship.

A visualization graph (**Figure 4A**) depicting collaboration relationships among journals reveals that, at present, there is no significant collaborative interaction between these academic publications. This suggests that the independent and distinct contributions are made by these journals in advancing the field of NAC for bladder cancer, with each journal fostering its own unique academic impact. In the dual-map overlay of journals and citing journals in **Figure 4B**, there were 2 main citation lines suggesting that the papers published on the journals of Medicine/Medical/Clinical could be cited by the papers on Health/Nursing/Medicine or Molecular/Biology/Genetic journals.

### Authors and co-cited authors

**Figure 5:**
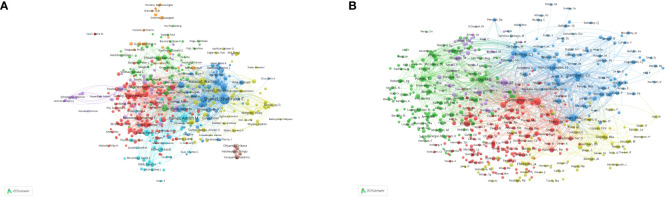
Analysis of authors and co-cited authors with a minimum of 5 citations. **(A)** The visualization map of authors analysis. **(B)** The visualization map of co-cited authors analysis. Each node represents an author or co-cited author, with node size indicating their citations or documents, and the lines between authors representing their collaborations.

**Table 4** presents the top 10 prolific authors and the top 10 co-cited authors with the highest citations in the domain of neoadjuvant chemotherapy (NAC) for bladder cancer. Shariat Shahrokh F, affiliated with the University of Vienna, distinguishes himself as the foremost contributor in this field. He claims the top position in the number of publications, total citations, and total link strength. This multifaceted achievement underscores his remarkable productivity, high-quality research output, and cooperative engagement with fellow authors. **Figure 5A** offers a visualized representation of co-author relationships, indicating the collaborative networks among authors. In our analysis, we did not discern any clear co-authored relationships, signifying that research contributions in this field have primarily been characterized by independent efforts.

**Table 4:** The 10 authors and the top 10 co-cited authors with the highest citations.

Rank	Author	Count	Total citations	Co-cited author	Total citations
1	Shariat Shahrokh F	95	5779	Grossman HB	972
2	Kamat Ashish M (BCG)	52	3519	Sternberg CN	779
3	Lotan Yair	50	4040	Herr HW	752
4	Kassouf Wassim	48	2708	Stein JP	719
5	Daneshmand Siamak	39	1113	Abol Enein H	713
6	Karakiewicz Pierre I	37	2909	Bellmunt J (ICI)	607
7	Galsky Matthew D	37	1447	Shariat SF	505
8	Lerner Seth P	37	3741	Galsky MD	503
9	Black Peter C	36	868	Von Der Masse H	488
10	Montorsi Francesco	35	1371	Witjes JA	481

By means of co-cited author analysis, we gain insight into the interconnections between authors who share similar research interests. Among these, Grossman HB, Sternberg CN, and Herr HW emerge as the top three co-cited authors. **Figure 5B** visually represents these relationships, with the number of lines connected to each author’s bubble corresponding to the extent of their co-cited connections. This visually highlights the considerable influence exerted by these authors within the field of NAC for bladder cancer.

### Co-cited references

**Table 5** provides a listing of the top 10 co-cited papers in the field of neoadjuvant chemotherapy (NAC) for bladder cancer. A noteworthy observation is that seven out of these top 10 co-cited papers were published during the period spanning 2000 to 2005. These papers reflect seminal contributions to the literature that continue to exert a significant influence. These top 10 co-cited references encompass a diverse range of publications, comprising three meta-analyses, five clinical trial articles, and a paper focusing on molecular subtypes of bladder cancer and guideline updates. Among them, the article titled “Neoadjuvant chemotherapy plus cystectomy compared with cystectomy alone for locally advanced bladder cancer”, published in the New England Journal of Medicine in 2003, emerges as the top co-cited reference, accumulating more than nine hundred citations.

**Table 5:** Top 10 co-cited references concerning the research of neoadjuvant chemotherapy for bladder cancer.

Rank	Journal	Author	Citations	Doi
1	New England Journal of Medicine	Grossman H B	933	10.1056/nejmoa022148
2	Clinical Oncology	Stein J P	484	10.1200/jco.2001.19.3.666
3	Clinical Oncology	Griffiths G	369	10.1200/jco.2010.32.3139
4	European Urology	Abol-Enein H	367	10.1016/j.eururo.2005.04.006
5	Clinical Oncology	Von Der Masse H	259	10.1200/jco.2000.18.17.3068
6	Lancet	Abol-Enein H	242	10.1016/S0140-6736(03)13580-5
7	Clinical Oncology	Von Der Masse H	200	10.1200/jco.2005.07.757
8	Cancer Cell	Choi W	181	10.1016/j.ccr.2014.01.009
9	European Urology	Bono A V	165	10.1016/j.eururo.2005.04.005
10	European Urology	Witjes J A	165	10.1016/j.eururo.2016.06.020

**Figure 6:**
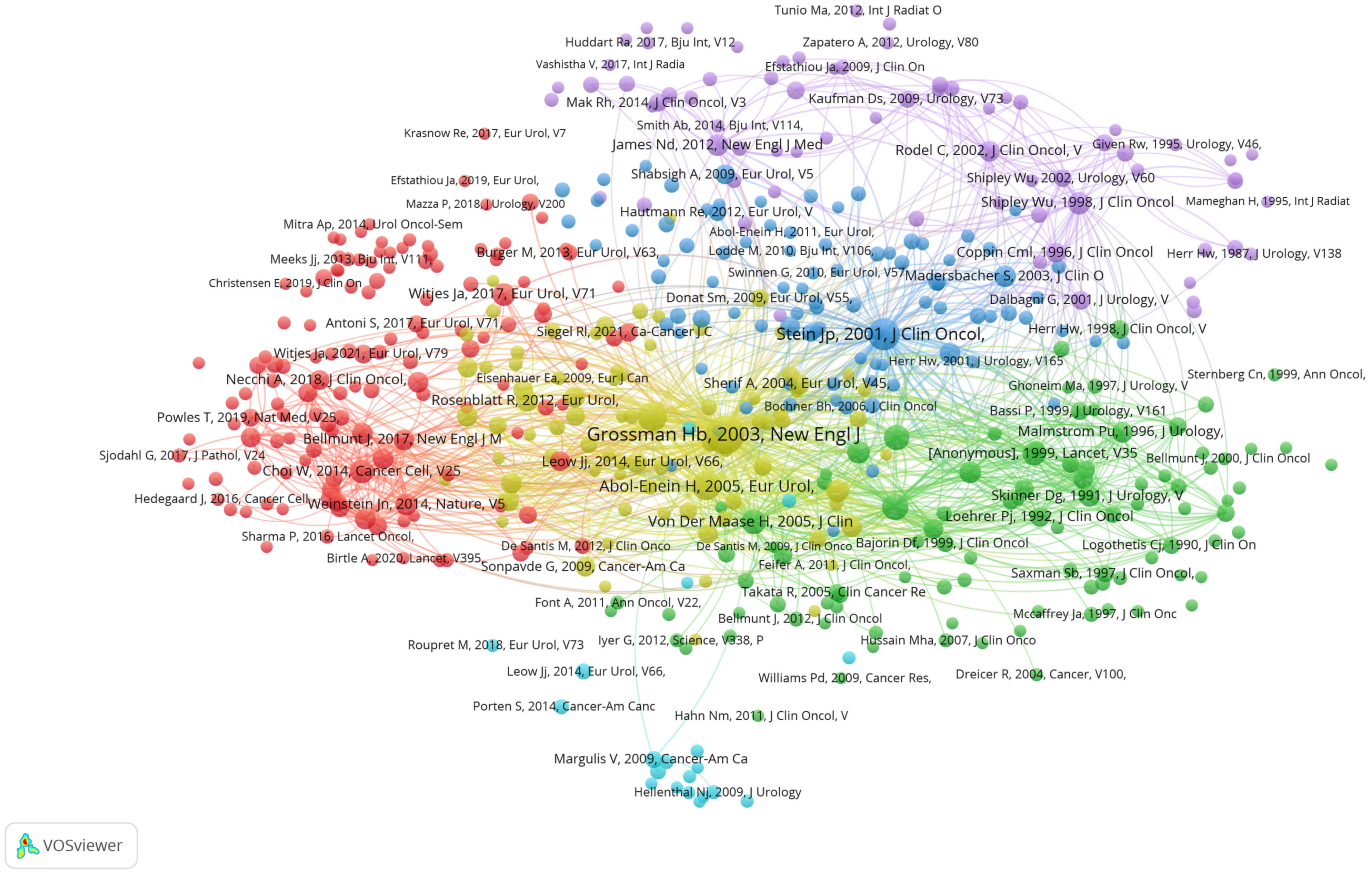
Visualization graph of co-cited references analysis with a minimum of 20 citations. the size of each bubble and the quantity of connected lines represent the TLS and correlations among co-cited references, respectively.

To further explore the relationships between these co-cited references, we employed a visualized graph of co-cited references analysis (**Figure 6**). Intriguingly, while the graph provides a holistic view of the interconnectedness of these references, it does not reveal any significant cluster trends, suggesting a broad and diverse body of literature with varying influences and interconnections.

### Keywords

**Figure 7:**
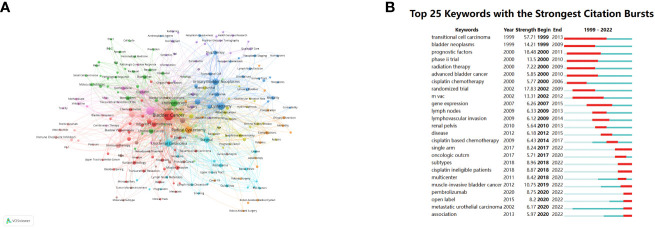
Visualization graph of keywords co-occurrence analysis with a minimum of 5 occurrence each node represents a keyword, with node size indicating their occurrence times, and the lines between keywords representing their collaborations **(A)**. **(B)** Top 25 keywords with the strongest citation bursts.

As depicted in **Figure 7A**, the visual representation generated using VOSviewer provides insight into the occurrence frequency and correlations among keywords. Among the top 5 keywords with the highest occurrence, “Bladder cancer”, “Neoadjuvant chemotherapy”, “Cystectomy”, “Chemotherapy” and “Radical cystectomy” are central to the discourse in this research domain, reflecting the core themes of investigation.

Furthermore, **Figure 7B** showcases the top 25 keywords characterized by the strongest citation bursts, effectively representing the evolving trends and hotspots within the field of NAC for bladder cancer over time. “Transitional cell carcinoma”, which represents the most common histopathological type in bladder cancer, demonstrates the highest citation strength and the longest duration, with a burst period spanning from 1999 to 2013 and a strength of 57.71. This extended burst period highlights the enduring significance of this keyword within the literature. Additionally, keywords such as “Bladder neoplasms”, “Prognostic factors”, “Phase II trial”, “Randomized trial”, “MVAC” and “Muscle-invasive bladder cancer” exhibit burst strengths exceeding 10, signifying their dynamic and influential roles as research topics and focal points in the field of NAC for bladder cancer. These burst keywords underscore the evolving nature of research interests and the emergence of new themes and research directions over time.

## Discussion

### General information

Multiple clinical trials indicate that exclusive reliance on surgery provides limited therapeutic advantages for MIBC patients. Conversely, neoadjuvant chemotherapy (NAC), especially those grounded in cisplatin-based regimens, is gaining prominence as a primary strategy, enhancing overall survival rates and mitigating recurrence and metastasis risks. Recent advances in NAC, including the integration of immunotherapy and targeted therapy, are transforming the treatment landscape for bladder cancer. This reflects a shift from traditional chemotherapy approaches to more personalized and effective strategies.

The field of neoadjuvant chemotherapy for bladder cancer has witnessed significant shifts in research focus over the years. Initially, studies predominantly focused on the efficacy and safety of platinum-based chemotherapy regimens. However, recent research has progressively embraced genetic markers and precision medicine, culminating in the latest trend towards immunochemotherapy approaches. These transitions reflect the dynamic adaptation of the field to emerging scientific insights and underline the critical importance of evolving treatment paradigms in response to these advances.

With the advent of innovative therapeutic agents, NAC research for bladder cancer has seen significant growth, substantially influencing MIBC patient management and prognosis. This growth is characterized by an expanded focus on molecular profiling and precision medicine, aiming to tailor treatments to individual patient needs and tumor characteristics. The surge in clinical trials aiming to ascertain the optimal NAC strategies and predictive indicators for patient outcomes has resulted in a plethora of publications. These trials underscore the necessity for ongoing research to refine NAC protocols and improve patient selection criteria.An in-depth analysis of the contributions from 57 countries or regions reveals a diverse research landscape. For instance, the United States, leading in publication volume, has fostered pioneering research in genomic predictors of NAC response. In contrast, European countries, with a focus on translational research, have contributed significantly to understanding the molecular mechanisms underlying bladder cancer. Institutions like the University of Texas and Memorial Sloan-Kettering Cancer Center have been instrumental in integrating clinical and translational research, demonstrating a holistic approach to NAC for bladder cancer. Regarding global contributions, the United States is at the vanguard of NAC research for bladder cancer, evidenced by its prolific publications, citations, and international collaborations. In contrast, European countries and China are emerging as significant contributors, each bringing unique perspectives and strengths to NAC research. Notably, six out of the ten leading contributors are European countries: Italy, Germany, the United Kingdom, France, Netherlands, and Austria. Although China, the sole developing country among the top ten, has exhibited exponential publication growth, it ranks second in the 2022 publication count. Yet, China’s citation metrics trail, suggesting a need for a more focused approach to enhance the quality and impact of its research contributions.

Prominent institutions, such as the University of Texas, Memorial Sloan-Kettering Cancer Center, and the University of Vienna, have garnered significant recognition. Their contributions, particularly in pioneering clinical trials and developing novel therapeutic approaches, have been instrumental in advancing NAC research.

In the journal spectrum, “European Urology” stands preeminent, boasting the superior Impact Factor among top-tier journals and underscoring its role as a primary conduit for NAC research dissemination. This prominence reflects the journal’s commitment to publishing pioneering studies that have significantly influenced the course of NAC treatment. Shahrokh F. Shariat of the University of Vienna has an exemplary publication record, notably eclipsing the second-ranked author, Ashish M. Kamat. Shahrokh F. Shariat’s research found in a phase 3, multicenter, double-blind, randomized, controlled trial that adjuvant nivolumab prolonged disease-free survival in patients with high-risk muscle-invasive urothelial carcinoma who had undergone radical surgery compared to placebo (21). This critical finding is dissected to showcase the evolving landscape of NAC where immunotherapeutic agents are becoming increasingly significant. The potential implications of these findings for future therapeutic strategies are also discussed.

Furthermore, HB Grossman’s research highlights the significance of cisplatin-based chemotherapy (M-VAC), which exhibits a superior median survival of 44 months in comparison to 37.5 months in the surgery group alone, and an overall 3-year survival rate improvement (22).These results emphasize the continued importance of traditional chemotherapy in NAC, while also highlighting the need for ongoing research to enhance efficacy and reduce toxicity.

Keyword analysis offers invaluable insights into predominant research foci, particularly evidenced by the surge in citations for terms like “Prognostic factors” and “Gene expression”. As therapeutic innovations emerge, clinical trials are increasingly pivotal, bridging foundational research and clinical application. While neoadjuvant immunotherapy presents promising potential, the keyword “Pembrolizumab” gained traction in 2020. Given the ongoing clinical trials, a surge in related keyword citations is anticipated. This anticipation is framed within the broader context of rapidly evolving NAC research, indicating a dynamic field that is responsive to new discoveries and treatment modalities. The potential for these trials to bring about significant shifts in the treatment landscape is explored, along with their implications for future research directions and clinical practice.

In summation, this exhaustive review elucidates the landscape of NAC research for bladder cancer, emphasizing global contributions, influential institutions, authors, journals, and emerging trends. Such insights empower researchers to adeptly navigate the domain, shaping future inquiries and enhancing bladder cancer patient outcomes.

Following this bibliometric analysis, several pivotal subtopics in neoadjuvant chemotherapy for bladder cancer have been discerned:

### Regimens of platin-based chemotherapy

Since the 1980s, cisplatin-based neoadjuvant chemotherapy has been utilized with the primary objective of improving surgical resection outcomes and overall survival (5). The prescription of this neoadjuvant chemotherapy to medically eligible MIBC patients has achieved broad clinical agreement (23–25). With time, the paradigm of chemotherapy regimens has dynamically transformed, reflecting the advent of innovative chemotherapeutic agents and becoming a focal point of comprehensive research and publications.

A seminal clinical trial aimed to contrast the therapeutic effectiveness of neoadjuvant methotrexate, vinblastine, doxorubicin, and cisplatin (MVAC) chemotherapy combined with cystectomy against cystectomy as a standalone treatment. The study showcased favorable outcomes, delineating a marked advantage in complete pathological response rates for patients undergoing neoadjuvant chemotherapy (38% vs. 15%, P < 0.001) (22). This noteworthy outcome underscored the preeminence of NAC, marking a significant milestone in the realm of NAC studies, as evidenced by its top-ranking in co-cited reference analysis presented in **Table 5**. Furthermore, alternative regimens, including cisplatin with methotrexate (CM), cisplatin with Adriamycin (CA), cisplatin combined with methotrexate and vinblastine (CMV), and the combination of gemcitabine and cisplatin (GC), have all affirmed their therapeutic potency (26–28). Building on the foundational contributions of urologists, numerous meta-analyses have furnished compelling evidence underscoring the advantages of neoadjuvant chemotherapy for patient prognosis, thereby cementing its role in clinical practice (29–31). The recent VESPER trial insights have enriched our comprehension of neoadjuvant chemotherapy, elucidating that dose-dense MVAC (dd-MVAC) surpasses the commonly adopted GC regimen in local tumor management and achieving superior three-year progression-free survival rates (32). In summation, the domain of neoadjuvant chemotherapy for bladder cancer is poised for further evolution, with prospective research endeavors targeting the formulation of holistic protocols that promise enhanced therapeutic outcomes, expansive applicability, and economic efficiency.

### Prognostic factors and biomarkers

Bladder cancer, characterized by its pronounced heterogeneity, presents clinical challenges, especially when implementing NAC for patients resistant to chemotherapy. The identification of prognostic factors, biomarkers, and appropriate patient cohorts remains paramount. The advent of advanced sequencing technologies and bioinformatics tools, notably the TCGA database, facilitates a comprehensive analysis of expansive genetic datasets spanning various bladder cancer subtypes.

The predictive function of molecular subtypes in relation to response rates remains unclear owing to the absence of a standardized approach in molecular categorization and the mechanism by which they do so remains unknown (33). As our understanding of the fundamental mechanisms of carcinogenesis expands, the link between gene mutations and resistance to chemotherapeutic agents becomes clearer, paving the way for the identification of new biomarkers. Several gene mutations, including ERCC2 (34), ERBB2 (35), and BRCA1 (36), are strongly correlated with NAC outcomes. In ATM/RB1/FANCC (37) mutant research, patients receiving cisplatin-based neoadjuvant chemotherapy for Muscle-invasive Bladder Cancer who possessed one or more ATM/RB1/FANCC mutations had 5-year survival rates that were higher for both OS (85% mutant vs. 46% wild) and DSS (90% mutant vs. 49% wild), respectively, than those without such mutations. Likewise, recent studies have indicated a positive correlation between deleterious mutations in ERCC2 and the pathological response to neoadjuvant chemotherapy based on Cisplatin. Patients who had ERCC2 mutations had a 5-year overall survival rate of 75%, while those who did not had that rate at 52% (38). Molecular pathology classifications further elucidate NAC responses. Utilizing immunohistochemical profiling to identify specific gene expressions, researchers have classified muscle-invasive bladder cancer into distinct molecular subtypes. Echoing molecular subtypes found in breast cancer, Choi et al. identified three unique bladder cancer subtypes, emphasizing the p53-like MIBC, known for its pronounced NAC resistance (39). This seminal study underscores the relationship between molecular subtypes and NAC responses. Building on the TCGA dataset, Robertson et al. identified five MIBC molecular subtypes: luminal-papillary, luminal-infiltrated, luminal, basal/squamous, and neuronal (40). While basal/squamous MIBC might respond positively to NAC, it could also be associated with an unfavorable prognosis. Several methods, including circulating tumor DNA (ctDNA) (41), urine tumor DNA (utDNA) (42) and clinical factors (43), have been explored to predict NAC responses. However, a substantial divide persists between experimental techniques and tools suitable for clinical application.

### Emerging trends in neoadjuvant chemotherapy for bladder cancer

Recent breakthroughs in immunotherapy have revolutionized the approach to neoadjuvant chemotherapy in bladder cancer (44).The introduction of immune checkpoint inhibitors (ICIs) such as pembrolizumab and atezolizumab has paved the way for new therapeutic combinations. Studies are increasingly focusing on the efficacy of combining ICIs with traditional cisplatin-based chemotherapy, revealing potential synergies that enhance response rates and improve survival outcomes compared to chemotherapy alone (45). This innovative approach presents a promising advancement in the treatment of bladder cancer, offering more personalized and effective therapeutic strategies.

Recent studies have also highlighted the potential of integrating genomic profiling into the NAC paradigm, offering a more personalized approach to treatment. For instance, research identifying specific molecular subtypes of bladder cancer that respond favorably to NAC opens new avenues for patient-specific therapy optimization. Additionally, ongoing trials exploring the combination of immunotherapy with NAC underscore the evolving nature of bladder cancer treatment and the necessity to adapt to these advancements. The integration of precision medicine into bladder cancer treatment is an emerging trend that warrants further exploration. Advances in genomic profiling and biomarker identification are enabling the development of targeted therapies tailored to individual patient profiles. Research into molecular biomarkers to predict response to neoadjuvant chemotherapy and immunotherapy is particularly promising (46). This direction may lead to the formulation of more effective and less toxic treatment regimens, optimizing patient care.Looking to the future, understanding the mechanisms of resistance to chemotherapy and immunotherapy in bladder cancer is a crucial research area. Investigating the genetic and molecular basis of resistance can uncover new therapeutic targets. Another burgeoning area of interest is the role of the tumor microenvironment in influencing bladder cancer progression and treatment response. Studies exploring the interactions between tumor cells, immune cells, and other microenvironment components could unveil novel methods to enhance the efficacy of neoadjuvant chemotherapy. This perspective is further reinforced by the findings of Zhou et al. (47), who explored temozolomide-based sonodynamic therapy and its role in inducing immunogenic cell death in glioma, an approach that might offer insights for bladder cancer treatment strategies (48). Their work exemplifies the kind of innovative approaches that could be pivotal in advancing neoadjuvant chemotherapy techniques.

In addition, exploring the potential of novel agents and combination therapies, especially in the context of drug resistance, is a critical area for future research. The development of therapies targeting specific molecular pathways implicated in bladder cancer progression, such as FGFR3 inhibitors and PARP inhibitors, holds promise for improving treatment outcomes (49).

### Future prospects

Patients who were administered neoadjuvant camrelizumab, in conjunction with gemcitabine and cisplatin, demonstrated a pathological response rate of 43.3% and a pathologic downstaging rate of 53.3% (50). These findings underscore the potential of incorporating novel ICIs into NAC regimens, potentially redefining treatment standards for bladder cancer. In contrast, the combination of rapamycin with gemcitabine-cisplatin did not show improved efficacy in treating muscle-invasive bladder cancer, as evidenced by a relatively low complete response rate of 23%, despite the inhibition of mTOR in tumor cells (51). This underscores the importance of ongoing clinical trials and research in identifying the most effective treatment combinations. The exploration of these novel therapies is critical to advancing our understanding of bladder cancer treatment and improving patient outcomes.

When patients receiving neoadjuvant platinum-based combination immune ICIs (ipilimumab plus nivolumab) for locally advanced urothelial cancer were compared with neoadjuvant platinum-based combination chemotherapy, the results indicated that patients receiving platinum-based combination ICIs achieved a greater degree of pathological complete pathological response rate (52). Although platinum-based regimens have shown benefits in tumor downstaging and improved long-term survival for certain patients, they also pose considerable risks and challenges. These include the potential for non-responsiveness in a subset of patients, significant toxicities, and the exclusion of patients with renal impairment. Understanding and mitigating these challenges are critical for maximizing the therapeutic potential of NAC in MIBC.

Furthermore, initially employed as a second-line treatment for advanced urothelial cancer, immunotherapy has shown significant therapeutic efficacy as a standalone neoadjuvant treatment, boasting an acceptable tolerability profile (53–55). The success of immunotherapy in these contexts highlights its potential as a transformative approach in NAC, offering new hope for patients with advanced bladder cancer.

Patients who have been diagnosed with metastatic urothelial carcinoma and are having chemotherapy that is based on platinum have a greater overall survival rate (median 12.8 months) in comparison to patients who are receiving immunotherapy (median 6.3 months) (56). In addition, a large-scale, randomized, international, multicenter Phase III Study was carried out in order to compare the efficacy of gemcitabine plus cisplatin (GC) with methotrexate, vinblastine, doxorubicin, and cisplatin (MVAC) for locally advanced or metastatic transitional-cell carcinoma patients. The findings revealed that overall survival was comparable between GC and MVAC, and the toxic mortality rate was 1% on the GC group as opposed to 3% on the MVAC group. Thus, GC gives a similar survival benefit to MVAC with a higher safety profile and tolerability (57). In our bibliometric analysis, we spotlight the increasing prominence of the keyword “Pembrolizumab” an “ICI”, which has seen a significant citation surge since 2020. This reflects a growing interest in the combination of immunotherapy with chemotherapy, especially considering the potential of cisplatin-based agents to potentiate the anti-cancer effects of ICIs by enhancing intratumoral T-cell activity (58). Recent studies suggest that ICIs, in conjunction with gemcitabine and cisplatin, yield positive results in terms of complete response rate and event-free survival (12, 59, 60). Ongoing trials, including EV304 and KEYNOTE-866, aim to ascertain the efficacy and safety of this combined approach.

In recent years, artificial intelligence (AI) has showcased its potential in predicting NAC outcomes in colorectal, breast, and gastric cancer (61–64). The application of AI in NAC for bladder cancer is still nascent, but significant strides have been made. For instance, predictive models for NAC responses have been formulated using machine-learning techniques based on biopsy pathology images (65). Similarly, Hepburn et al. utilized machine learning to pinpoint novel NAC predictive biomarkers (66). While literature on AI’s role in NAC for bladder cancer remains sparse in our analysis, the future likely holds a significant role for AI in advancing NAC research, propelled by advancements in computational capabilities, algorithms, and clinical databases.

In conclusion, the integration of these clinical and translational breakthroughs into standard clinical protocols is imperative. It involves tailoring treatments to individual patients, enhancing the likelihood of complete responses, and championing bladder preservation strategies. The future of NAC in bladder cancer treatment is poised at an exciting juncture, with the potential to significantly improve patient outcomes through innovative, personalized, and multidisciplinary approaches.

### Limitations

This analysis is not without limitations, primarily stemming from the exclusive reliance on the WOSCC database and the constraint to English-language publications. Additionally, we acknowledge that our analysis did not differentiate between subtypes of bladder cancer, such as urothelial and non-urothelial carcinomas, or between muscle invasive and non-muscle invasive forms. This broad approach may overlook specific trends within these distinct subgroups. Future endeavors should contemplate a more holistic approach, incorporating diverse data sources and languages.

## Conclusion

This bibliometric analysis has provided a comprehensive overview of NAC research in bladder cancer from 1999 to 2022, revealing the pivotal role of the United States and other regions in advancing the field. Importantly, this study highlights not just the evolution of chemotherapy regimens and the role of prognostic biomarkers, but also the emerging complexity within NAC treatment strategies. The findings underscore the growing importance of integrating immunochemotherapy and leveraging artificial intelligence (AI) to address the multifaceted challenges in bladder cancer treatment. Our analysis suggests a trend towards more integrated, personalized approaches, driven by a deeper understanding of the molecular and clinical complexities of bladder cancer. Future research should focus on further unraveling these complexities, particularly in optimizing immunochemotherapy strategies and utilizing AI for advanced patient stratification and treatment personalization. Such avenues are crucial for the continued advancement of bladder cancer treatment and improving patient outcomes, reflecting a deeper comprehension of the intricate interplay of various therapeutic modalities in NAC.

## Data availability statement

The original contributions presented in the study are included in the article/supplementary material. Further inquiries can be directed to the corresponding authors.

## Author contributions

YH: Conceptualization, Writing – original draft, Formal analysis, Investigation, Methodology. CL: Software, Writing – original draft, Formal analysis. ZS: Investigation, Conceptualization, Writing – review & editing. YZ: Data curation, Methodology, Writing – review & editing. WX: Conceptualization, Methodology, Writing – review & editing. QG: Formal analysis, Investigation, Software, Writing – review & editing. YY: Data curation, Investigation, Software, Writing – review & editing. JZ: Project administration, Supervision, Validation, Writing – original draft, Writing – review & editing. JK: Formal analysis, Methodology, Software, Supervision, Validation, Writing – original draft, Writing – review & editing.
